# Fabrication and Characterization of Chitosan—Tamarind Seed Polysaccharide Composite Film for Transdermal Delivery of Protein/Peptide

**DOI:** 10.3390/polym13091531

**Published:** 2021-05-10

**Authors:** Rishabha Malviya, Anchal Tyagi, Shivkanya Fuloria, Vetriselvan Subramaniyan, Kathiresan Sathasivam, Sonali Sundram, Sundram Karupiah, Srikumar Chakravarthi, Dhanalekshmi Unnikrishnan Meenakshi, Nandan Gupta, Mahendran Sekar, Kalvatala Sudhakar, Neeraj Kumar Fuloria

**Affiliations:** 1 Department of Pharmacy, SMAS, Galgotias University, Greater Noida, Gautam Budha Nagar 201310, India; rishabha.malviya@galgotiasuniversity.edu.in (R.M.); tyagianchal1234@gmail.com (A.T.); sonaliaim13@gmail.com (S.S.); nandangupta28@gmail.com (N.G.); 2 Faculty of Pharmacy, AIMST University, Bedong 08100, Malaysia; sundram@aimst.edu.my; 3 Faculty of Medicine, Bioscience and Nursing, MAHSA University, Kuala Lumpur 42610, Malaysia; drvetriselvan@mahsa.edu.my (V.S.); srikumar@mahsa.edu.my (S.C.); 4 Faculty of Applied Science, AIMST University, Bedong 08100, Malaysia; skathir@aimst.edu.my; 5 College of Pharmacy, National University of Science and Technology, Muscat 130, Oman; dhanalekshmi@nu.edu.om; 6 Department of Pharmaceutical Chemistry, Faculty of Pharmacy and Health Sciences, Universiti Kuala Lumpur Royal College of Medicine Perak, Ipoh 30450, Malaysia; mahendransekar@unikl.edu.my; 7 School of Pharmaceutical Sciences (LIT-Pharmacy), Lovely Professional University, Jalandhar 144411, India; sudhakar.20477@lpu.co.in

**Keywords:** transdermal film, drug delivery, peptide delivery, Hixson-Crowell kinetics

## Abstract

Transdermal drug delivery is used to deliver a drug by eliminating the first-pass metabolism, which increases the bioavailability of the drug. The present study aims to formulate the chitosan—tamarind seed polysaccharide composite films and evaluate for the delivery of protein/peptide molecules. Nine formulations were prepared and evaluated by using different parameters, such as physical appearance, folding endurance, thickness of film, surface pH, weight variation, drug content, surface morphology, percentage moisture intake and uptake, drug release kinetics, and drug permeability. The film weight variance was observed between 0.34 ± 0.002 to 0.47 ± 0.003 g. The drug level of the prepared films was found to be between 96 ± 1.21 and 98 ± 1.33μg. Their intake of moisture ranged between 2.83 ± 0.002 and 3.76 ± 0.001 (%). The moisture absorption of the films ranged from 5.33 ± 0.22 to 10.02 ± 0.61 (%). SEM images revealed a smooth film surface, while minor cracks were found in the film after permeation tests. During the first 4 days, drug release was between 13.75 ± 1.64% and 22.54 ± 1.34% and from day 5 to day 6, it was between 72.67 ± 2.13% and 78.33 ± 3.13%. Drug permeation during the first 4 days was 15.78 ± 1.23 %. Drug permeation (%) during the first 4 days was between 15.78 ± 1.23 and 22.49 ± 1.29 and from day 5 to day 6, it was between 71.49 ± 3.21 and 77.93 ± 3.20.

## 1. Introduction

A lot of bioactive proteins/peptides have been used as medical therapeutics in last decades. Advancement in biotechnology and developments in the chemistry of peptides resulted in syntheses of bioactive peptide molecules. When administered orally, these biomolecules get degraded in the gastrointestinal tract or conformational changes may take place [1]. Even in an aqueous environment, during high-speed agitation, proteins can be easily denatured, precipitated, or aggregated. Oral delivery of therapeutic peptide molecules is hampered by their intrinsic properties [1]. These limitations motivate researchers worldwide to develop alternative routes for peptide delivery. To overcome these limitations, peptide molecules are generally administered by injection or through skin [2]. Parenteral administration is an invasive route and has some limitations and lower patient compliance. Transdermal administration of peptide moiety avoids needle phobia and is thus a preferable route for delivery [2]. Transdermal delivery of drugs also eliminates first-pass metabolism and, hence, improves drug bioavailability. Peptide delivery through transdermal route also improves drug concentration in blood for longer periods of time, especially for candidates having a short half-live. Peptides have limitless possibilities as therapeutics. Traditionally, a transdermal film is used to deliver drug candidates and not peptides [2]. As discussed by Alkilani et al., the transdermal route provides a uniform drug delivery and maintains the pharmacokinetic parameters unchanged. Transdermal film formulation of peptide molecules is an economical, easily accessible, non-invasive, and feasible strategy for drug delivery [3].

Body tissues and organ structures are diverse and respond to intrinsic changes during medical procedures. To provide an optimal drug delivery system, the biological tissue activities must be taken into consideration to develop formulations that can change their properties over time based on the requirements of the patient. Multitherapy and on-demand treatments need to be developed urgently in order to improve medicinal supply and bioavailability. Several “smart” drug delivery systems have been designed to achieve more effective therapies by offering new properties and new characteristics that can respond to different environmental challenges. Polymeric biomaterials offer great opportunities for increasing drug biodistribution in different biomedical applications (e.g., cancer or antibacterial therapy).

In certain biomedical applications (e.g., tissue engineering and biosensing), stimuli-responsive materials are used and are regarded as possible candidates for pulsatile, site-specific, and externally induced drug release systems. Hydrogels have received great interest among this group of materials because of their tuneable characteristics, including mechanical strength and structural, chemical, and biological responses to stimuli. A nano-fibre nonwoven fabric is an additional component of our “smart” drug distribution systems. Polymeric electrospun nanofibers are a recent desirable candidate for tissue engineering the drug delivery [4,5].

Hydrophilicity and molecular size both limit the transdermal delivery of therapeutic peptides. As a breakthrough in the pharmaceutical industry, new drug delivery methods enable to regulate the rate of drug delivery, preserve the time of therapeutic action, and more intensively target the drugs at particular sites, thereby giving birth to a new system of drug delivery [6]. In recent decades, a variety of new polymers have been prepared, which can react to pH, temperature changes, and electric or magnetic fields in any desired manner. Polysaccharide composite films are involved in the processing of therapeutic agents into non-deleterious forms that can be effectively prescribed, as well as directly influence the bio-distribution and cellular internalization, and are thus becoming increasingly important in biomedical science [7].

Progress in polysaccharide composites formation provides unique opportunities to formulation scientists to tune up the desired therapeutic effect. Polysaccharide composites are commonly and widely used in the production and design of controlled and sustained release formulations for this purpose [7]. They are provided as covering substances, drug carriers, granulating agents, tablet excipients (as binders, disintegrants, and fillers), film foaming agents, and solubilising agents due to their physicochemical properties. In recent years, across biomedical applications, such as wound cure, electrical equipment, and nanomedicine, protein/polysaccharide composites play an important part [8]. Improvements have been made in these processes by integrating the protein/polysaccharide composites in the use of hydrogel products to provide cartilage defects, electrospinning to develop antimicrobial properties for the treatment of wounds, and films for food packing and medication delivery. A typical composite protein content includes silk, keratin, soy, collagen, gelatine, resilin, zinc and wheat gluten [9].

Polysaccharides have several benefits over protein molecules, as they are normally more resilient and usually are not heat-inactivated. The wide variety of polysaccharides enables production of materials with small, medium, and large molecular weights and different polydispersion indices [10]. The distinct polydispersions are responsible for changes in structure, solubility, and toxicity of polysaccharides. Composites of polysaccharides and proteins have become widespread in the biomedical field because of the intermolecular interactions with the matrices which can form scaffolds, particles, films, fibres, and gels [11]. The structure of these composites makes it possible to improve the material properties of a protein by combining it with a polysaccharide [12].

Chitosan (CS) has been found to be advantageous over other polymers in case of PEC formation because its production avoids organic solvents and chemical cross-linking agents, so it can reduce toxicity and undesirable side effects. It is a very plentiful, environmentally safe, and suitable vehicle for the delivery of sustained medications [13]. Chitosan is a cationic natural biopolymer, biocompatible, non-antigenic, and non-toxic. Due to its good mechanical characteristics—biodegradability, biocompatibility, multiple functional groups, and solubility in aqueous medium—chitosan has been extensively studied for many decades for molecular separation, as a food packaging film, as an artificial skin, as a bone substitute, in water engineering, and so on [14]. However, its applicability is limited by its lack of thermal stability, hardness, and gas barrier properties. The chemical makeup of CS with many functional groups (hydroxyl, carbonyl, carboxyl, amine, amid) makes it possible to bind new particles—including clay (bentonite, montmorillonite), silica (SiO_2_), and carbon nanotubes—in the CS chain. The composite particles that are formed can be used for protection or delivery of a pharmaceutical or a nutrient, such as a drug or a bioactive lipid [15]. Chitosan’s versatility and application ranges in biomedical and other industrial sectors are highly sophisticated. Because of its cationic nature and its main amino group, chitosan has an advantage over other polysaccharides.

Skin is the human body’s largest organ. It is the first protection against environmental and surrounding threats for humans. Fabrication of a quaternary composite scaffold with chitosan, alginate, gelatine, and silk fibroin has successfully achieved 88% porosity with strong mechanical stability [16].

Tamarind (*Tamarindus indica*) is a leguminous tree in the Fabaceae family. Seeds of tamarind with a molecular weight between 720 and 880 kDa consist of up to 72 % mucilage. It is composed of a polysaccharide called xyloglucan, which is classified as hemicellulose and contains glucose, xylose, and galactose units [17].

Fabrications of polysaccharide-based composites containing different kinds of reinforcements were subjected to many applications. Due to their chemical composition and easy manipulation, polysaccharide-based composites are attractive biomaterials. They are readily processable and diverse in nature [17]. In contrast to other synthetic biomaterials, they are of large economic use. In addition, they are environmentally friendly and non-toxic to humans and wildlife.

Hydrophilicity and molecular size limit the transdermal delivery of therapeutic peptides. The primary challenging problem in protein delivery through the transdermal route is to consider the biological factor (skin conditions). In this study, egg membrane was used as a model biological barrier to mimic the skin. We attempted to analyse the efficacy of a chitosan–TSP polysaccharide composite film to deliver a model protein molecule (human serum albumin) through skin. Egg membrane was used as biological barrier to simulate skin.

## 2. Materials and Methods

### 2.1. Factorial Design 

Films were fabricated using 32 factorial design. The concentrations of TGP and Ct were chosen as independent variables in the current study, and T50% and T80% were the dependent variables. Three thresholds for each independent variable were chosen, as shown in Table 1. The effects of dependent variables were evaluated using the NCSS software (trial version 15/05/2018) [18].

### 2.2. Method of Preparation of Polysaccharide Composite Films

Composite films were prepared by a method described by Meng et al. with slight modifications. Using different ratios of tamarind gum polysaccharide (TGP) and Chitosan (Ct) with the model compound, composite-based films were prepared. To prepare the solution, various amounts of gum and chitosan were combined and stirred for 10 min at a temperature of 40 °C. The bovine serum albumin was applied to the polysaccharide composite solution after 24 h. It was kept at a temperature of 45 °C for 15 min for stirring and then poured into a mould and dried at 40 °C. The quantities of TGP and Ct used for the preparation of films are shown in Table 1.

### 2.3. Characterization of Films

#### 2.3.1. Physical Appearance

All the prepared film formulations were visually observed for any flaws.

#### 2.3.2. FTIR Spectral Analysis

Spectral analyses of the tamarind seed polysaccharide, chitosan, albumin, and formulated film were carried out to identify any possible interaction between the film components. The OPUS software was used to record spectra of all the compounds in the range of 4000–400 cm^−1^.

#### 2.3.3. Folding Endurance

Folding endurance is important to evaluate film strength. A specific area of film (1 cm × 1 cm) was sliced and folded until it was broken. The significance of folding endurance was provided by the number of times the films could be folded without fracturing [18].

#### 2.3.4. Thickness of Films

An ideal film should have a uniform thickness. The thickness of the prepared films was determined (in mm) at five different points using a screw gauge and the average with standard deviation was calculated [19,20].

#### 2.3.5. Surface pH

We hydrated the films by bringing them in contact with distilled water for a few minutes. The surface pH was then determined with a pH strip. Surface pH of a film is important to evaluate its irritant effect on the application site.

#### 2.3.6. Weight Variation

By independently weighing 1 cm × 1 cm of randomly chosen films, the films were subjected to weight variance assessment and the average with standard deviation was calculated [21].

#### 2.3.7. Drug Content

Pieces of 1 cm^2^ of film formulations were dissolved in a phosphate buffer (pH 6.8) and stirred adequately with a magnetic stirrer for 24 h. The percentage of drug content was measured spectrophotometrically at a wavelength of 202 nm after filtration and dilution with a phosphate buffer [22]. Drug content is defined as the quantity of drug present in the formulation. The drug quantity was expressed by the Equation (1).

Drug content = Concentration × Dilution factor(1)

#### 2.3.8. Moisture Intake

The prepared films were individually weighed and placed in a desiccator containing calcium chloride for 24 h at 35 °C. The films were reweighted after 24 h and the percentual humidity uptake was determined using the following Equation (2) [23].

Moisture intake (%) = (Initial weight − Final weight)/(Initial weight) (2)

#### 2.3.9. Moisture Uptake

The prepared films were weighed individually and placed in potassium chloride-containing desiccators for 24 h at 35 °C. The films were reweighted after 24 h and the percentage of moisture absorption was calculated by using the following Equation (3) [24].

Moisture intake (%) = ((Final weight − Initial weight)/ (Initial weight)) (3)

#### 2.3.10. Surface Morphology

SEM is primarily used for determination of morphology. Morphology of the films was analysed by the Zeiss EVO 18 analyser.

#### 2.3.11. Drug Release

The drug release of different formulations—i.e., films—was assessed using the eggshell membrane as a biological barrier.

##### Preparation of an Eggshell Membrane

A chicken egg was taken for the preparation of the eggshell membrane. An orifice was created at one end of the egg and the yolk was removed through the opening. The eggshell was placed in a beaker containing acidic water. The beaker was equipped with a heating mantel to maintain 45 °C. We waited until the bubbling ends and the foam faded [25]. As calcium carbonate was released as foam from the eggshell upon contact with HCl, only the membrane remained in the beaker.

##### In Vitro Drug Release

Pieces of 1 cm^2^ of films were held and bound properly in the isolated egg membrane. The drug release was measured for 5 days in IP phosphate buffer (pH 6.8). The temperature of the medium was maintained at 37 ± 0.5 °C at a constant stirring speed of 50 rpm. A volume of 5 mL of the sample was isolated from the main solution at a set time interval, and 5 mL of the buffer was restored at the same time interval. Sample processing was carried out at a wavelength of 202 nm using a UV spectrophotometer [26]. The following Equation (4) was used to determine the drug release %.

Drug release % = (Concentration of formulation × Dilution factor × volume of dissolution medium)/1000 × 100 (4)

#### 2.3.12. Kinetics of Drug Release

Determination of the dissolution profile of a formulation is often necessary. The study of kinetics was chosen to find out whether the dissolution took place properly [27]. Kinetics models are used to assess the dissolution of a drug, in the mixture of which the amount of the dissolved drug is determined as a function of time. Using a variety of modal kinetics, the release of drug formulations may be represented [28]. Zero order, first order, Hixson–Crowell, Higuchi, Baker–Lonsdale, and Korsmeyer–Peppas models were applied in the kinetics study [29]. 

In this case, we observed that the Hixson–Crowell model corresponded to the kinetics of this formulation. The Hixson–Crowell model can be used when the surface area and diameter of the drug matrix change in time. The Hixson–Crowell model data obtained from in vitro drug release studies were plotted as cube root of the drug versus time [30]. 

Hixson–Crowell rate constant can be determined using Equation (5) [30]:

W_0_ ^1⁄3^ − W_t_^1⁄3^ = K_HC_t (5)

where

W_0_ is the initial amount of drug in the dosage form
W_t_ is the remaining amount of the drug in the dosage form at time t
K_HC_t is a constant incorporating the surface volume relation.

#### 2.3.13. In Vitro Skin Permeation Studies

To assess in vitro drug diffusion from films, a modified Franz diffusion cell was used. The diffusion cell had a diffusion zone of 2.52 cm^2^ and a receptor volume of 20 mL. In the receptor compartment, IP phosphate buffer (pH 6.8) was kept as a dissolution medium. It was stirred at 300 rpm using a magnetic stirrer at a temperature of 37 ± 0.5 °C. Chicken abdominal membrane was used as a biological barrier to evaluate the diffusion of drugs through the membrane. Animal abdominal membrane had been used previously as an ex vivo model for transdermal drug delivery [31,32].

The used membrane mimicked functionalities of skin. A fixed size film (1 cm × 1 cm) was kept in the donor compartment. Aliquots were collected at various times and replaced with fresh buffer [33]. Drug permeation was determined by using a UV spectrophotometer (UV-1800, Shimadzu, Japan).

## 3. Results and Discussion

As described by authors in different studies, composite materials are combinations of two or more materials, exhibiting enhanced physicochemical properties compared to the individual materials [34]. Transdermal films of chitosan-based polysaccharides composite have been successfully prepared. Physicochemical evaluation of composite transdermal films included an evaluation of thickness, drug content, weight variation, moisture content, moisture absorption, swelling, IR spectra, and mechanical properties [35]. The result of moisture content measurement was in accordance with a study performed by Thakur et al., showing an average moisture content between 3.08% and 2.44%. Raman Jot Kaur et al. also obtained similar results; their measured the drug content of different batches of transdermal films to be between 95.81 ± 0.75% and 98.08 ± 0.88%. Salcedo et al. and Depan et al. also published similar findings about reduction in swelling capacity and water uptake profile [36,37]. Most natural polysaccharide-based materials have similarities to materials found in living organisms. These include the size of materials, which is usually 100 nm and below. Due to that, many natural polysaccharide-based composites can have multifunctional purpose in biological activities similar to cellular functions. The evaluation of composite transdermal films and their various physicochemical properties, as well as their characterization by IR spectroscopy, drug release studies, and stability testing are discussed hereunder [34,35,36,37].

Polysaccharide-based composite film formation opens a new area of research and gains significant interest worldwide for the delivery of potential active therapeutic agents.

### 3.1. Factorial Design

Rao et al. have prepared a transdermal patch of ketoprofen and its optimization was done using 32 factorial design method, which is in accordance with our proceeding [38]. In the present study, 32 factorial design was used to evaluate the effect of independent variables (T50% and T80%) of film (dependent variable). A reduced equation to measure a response (T50% and T80%) having statistical significance for the 32 factorial design is shown here (Equation (6)).

(6) $$y=b_{0}+b_{1}x_{1}+b_{2}x_{2}+b_{12}x_{1}x_{2}+b_{11}x_{12}b_{22}x_{22}$$

where

Y = response of variables (dependent variables)
b_0_ = arithmetic mean response of nine batches
b_1_ = estimated coefficient of factor X_1_. The coefficients corresponding to the liner effect (b_1_ and b_2_), interaction (b_1_), and quadratic effect (b_1_ and b_2_) were determined from the result of the experiment.
X_1_ and X_2_ are quantities of the polymers.

Based on our experimental findings, Equation (6) was solved to calculate the effect of polymer quantity over T50% (Equation (7)) and T80% (Equation (8)) of films.

(7) $$T_{50}\;\left(h\right)=195.88-2.11\left(X_{1}\right)+0.22\left(X_{2}\right)-2.11\left(X_{1}X_{2}\right)+0.629\left(X_{12}\right)-1.48\left(X_{22}\right)$$

(8) $$T_{80}\;\left(h\right)=205.77-2.22\left(X_{1}\right)+0.11\left(X_{2}\right)-2.22\left(X_{1}X_{2}\right)+0.592\left(X_{12}\right)-1.51$$

**Figure 1:**
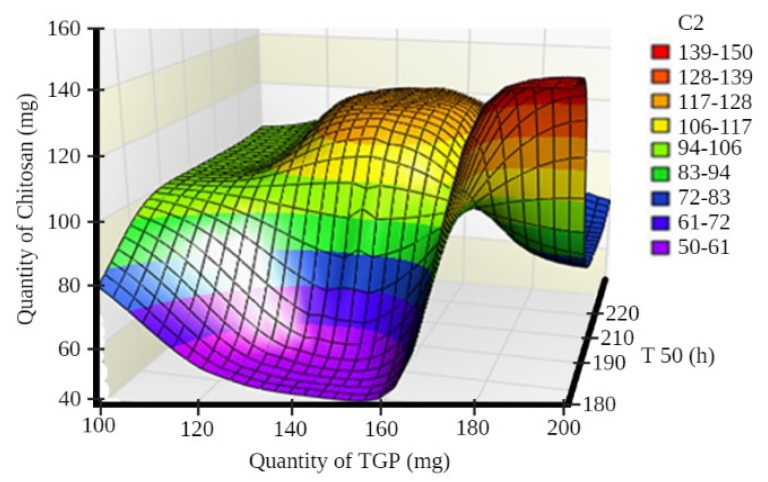
Surface plot showing the effects of independent variables of films.

**Figure 2:**
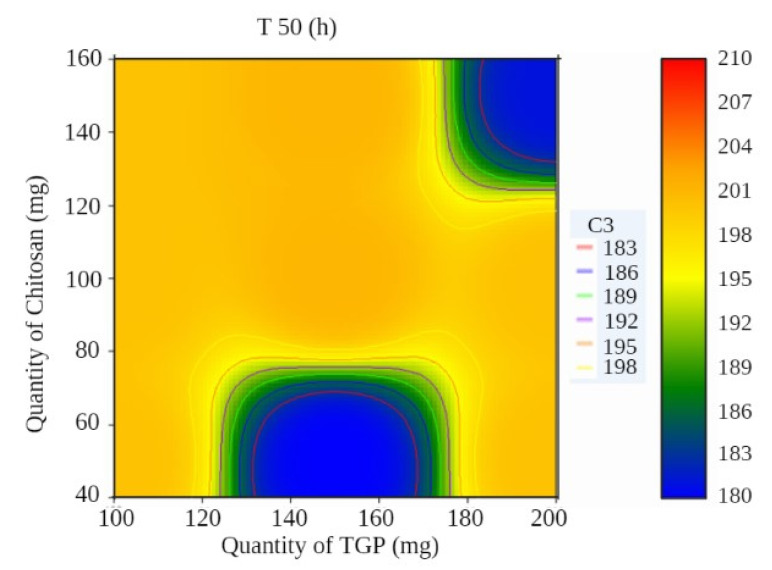
Contour plot showing the effects of independent variables of films.

A surface plot and contour plot showing the effects of independent variables of the films—TGP, chitosan, and T50%—are shown in Figure 1 and Figure 2, respectively.

In surface response curve, “C2” and in contour plot “C3” represents colour band to identify response (dependant variables) of the study.

**Figure 3:**
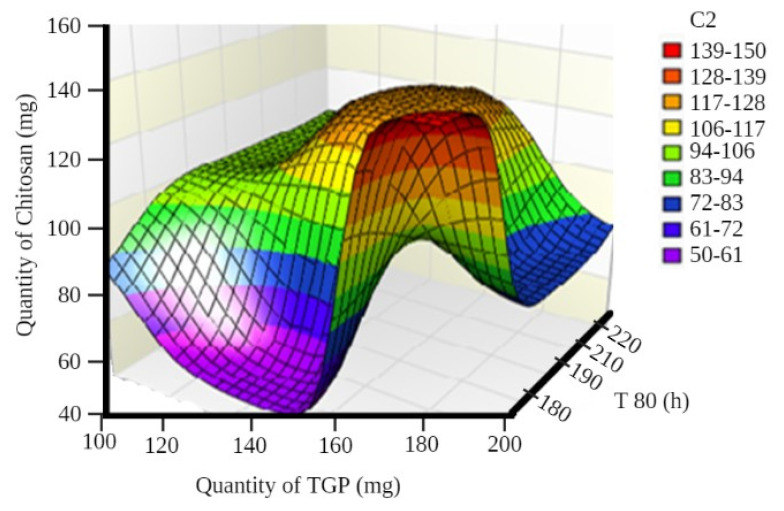
Surface plot showing the effects of independent variables of films.

**Figure 4:**
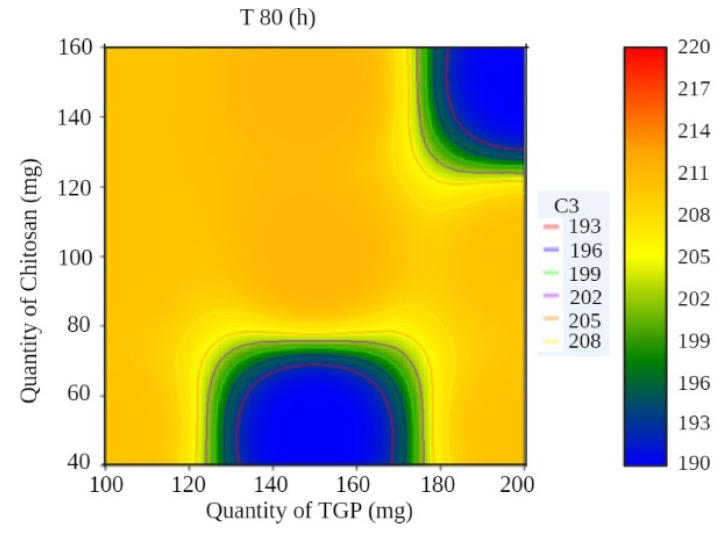
Contour plot showing the effects of independent variables of films.

A surface plot and contour plot showing the effects of independent variables of the films—TGP, chitosan, and T80%—are shown in Figure 3 and Figure 4, respectively.

Based on our experimental findings, Equation (6) was solved to calculate the effect of polymer quantity over T50% (Equation (9)) and T80% (Equation (10)) of films.

(9) $$T_{50}\left(h\right)=200.44+0.22\left(X_{1}\right)+0.22\left(X_{2}\right)-0.11\left(X_{1}X_{2}\right)-0.074\left(X_{12}\right)-0.074\left(X_{22}\right)$$

(10) $$T_{80}\left(h\right)=210.44-0.22\left(X_{1}\right)+0.22\left(X_{2}\right)-0.11\left(X_{1}X_{2}\right)+0.074\left(X_{12}\right)-0.074\left(X_{22}\right)$$

**Figure 5:**
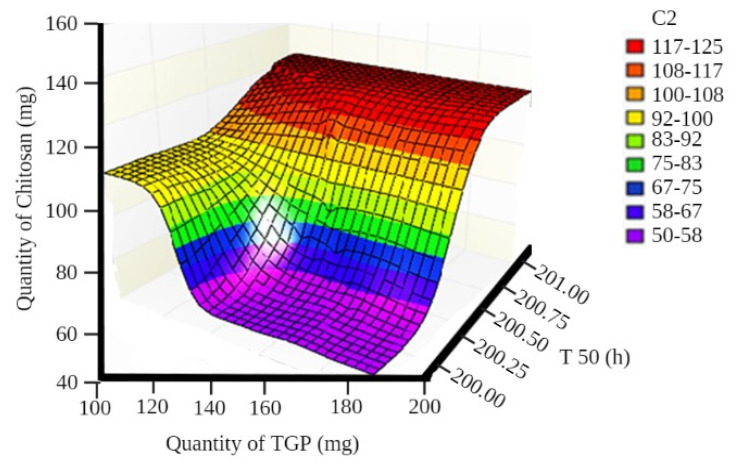
Surface plot showing the effects of independent variables of films.

**Figure 6:**
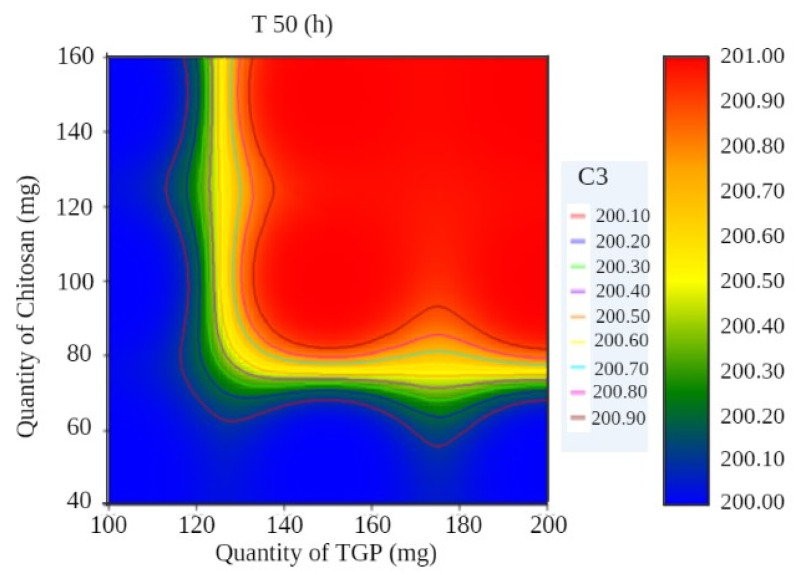
Contour plot showing the effects of independent variables of films.

**Figure 7:**
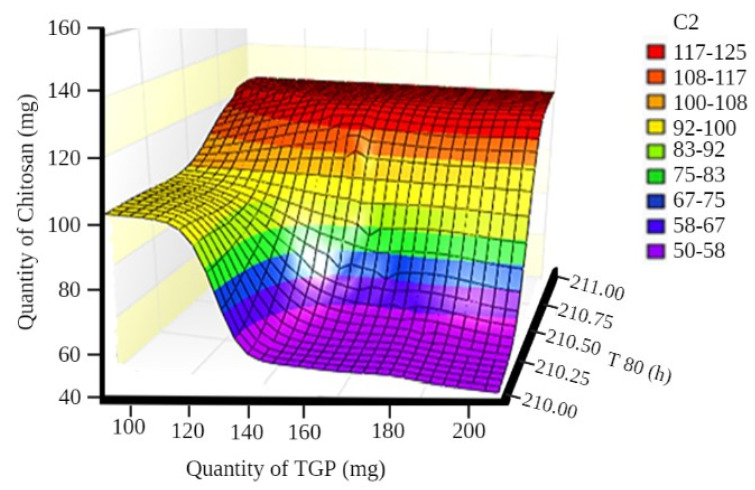
Surface plot showing the effects of independent variables of films.

**Figure 8:**
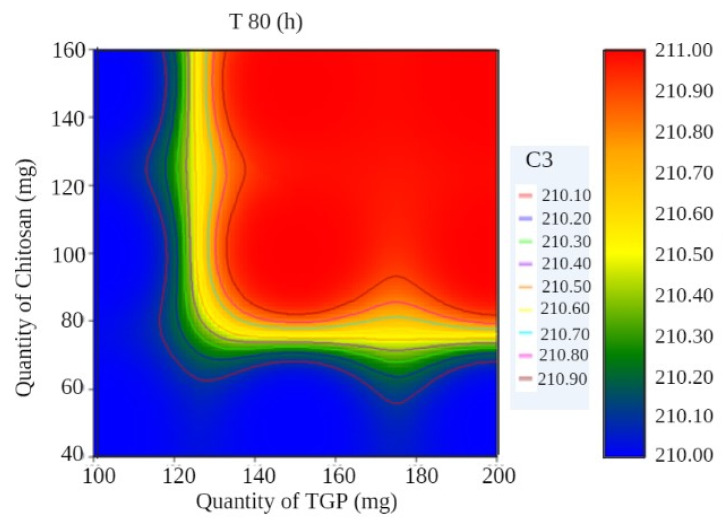
Contour pot showing the effects of independent variables of films.

Surface plots and contour plots showing the effects of independent variables of the films—TGP, chitosan, and T50%—are shown in Figure 5, Figure 6, Figure 7 and Figure 8.

### 3.2. Physical Appearance

It was observed that the whole prepared film structure was opaque and translucent. Zhang et al. have also prepared a transparent amorphous cellulose film, in accordance with our results [39].

### 3.3. IR Spectral Analysis

**Figure 9:**
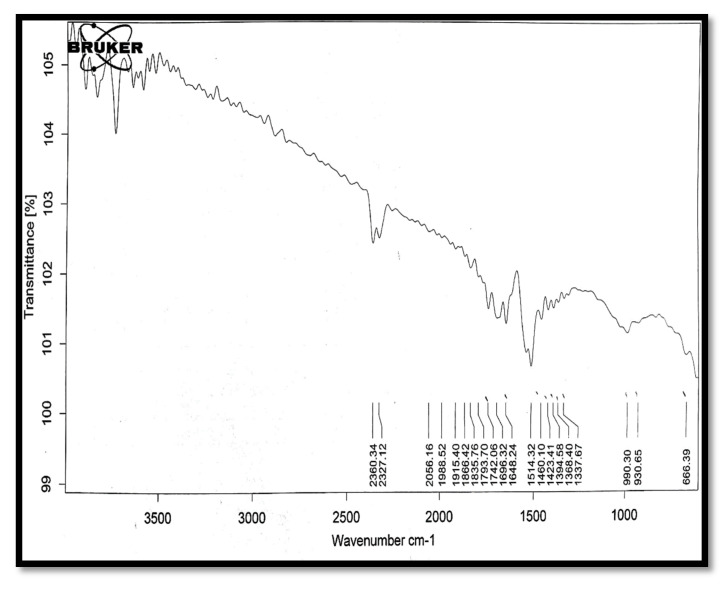
The IR spectrum of tamarind gum polysaccharide.

In the infra-red (IR) spectrum of tamarind gum polysaccharide (Figure 9), peaks of O–H stretch (3616 cm^−1^), COOH (2361 cm^−1^), C≡C stretch (2056 cm^−1^), C=C stretch (1648 cm^−1^), and C–O (990 cm^−1^) were present.

**Figure 10:**
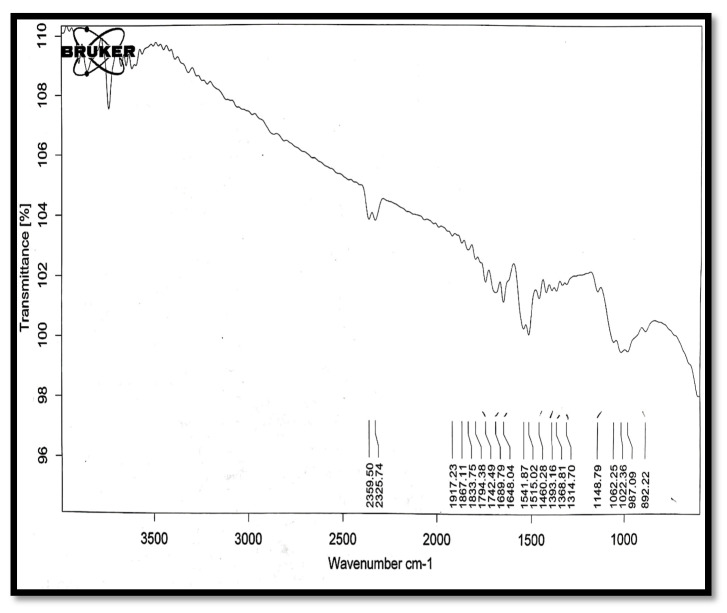
The IR spectrum of chitosan.

In the spectrum of chitosan (Figure 10), peaks of C≡N stretch (1648 cm^−1^), COOH (2360 cm^−1^), and O–H (3616 cm^−1^) were present.

**Figure 11:**
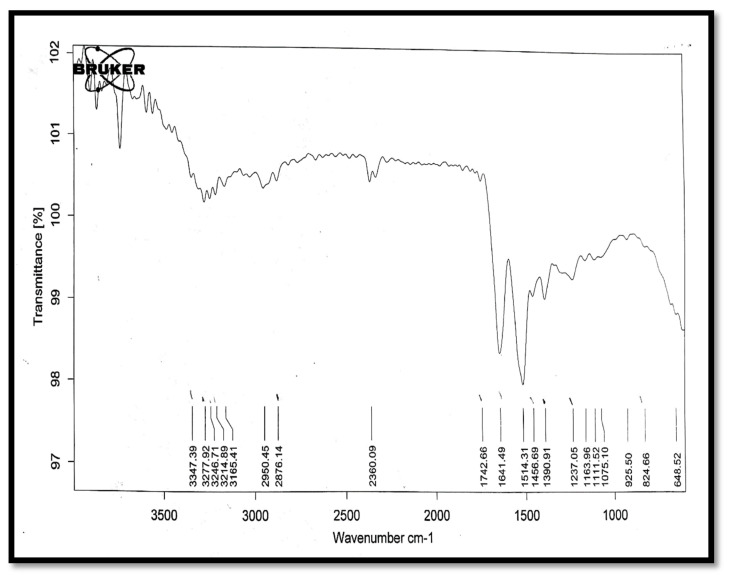
The IR spectrum of bovine serum albumin.

In the spectrum of bovine serum albumin (Figure 11), peaks of C≡N stretch (1590 cm^−1^), C–Cl stretch (639-cm^−1^), O–H (3347 cm^−1^), and S=O (1238 cm^−1^) were present.

**Figure 12:**
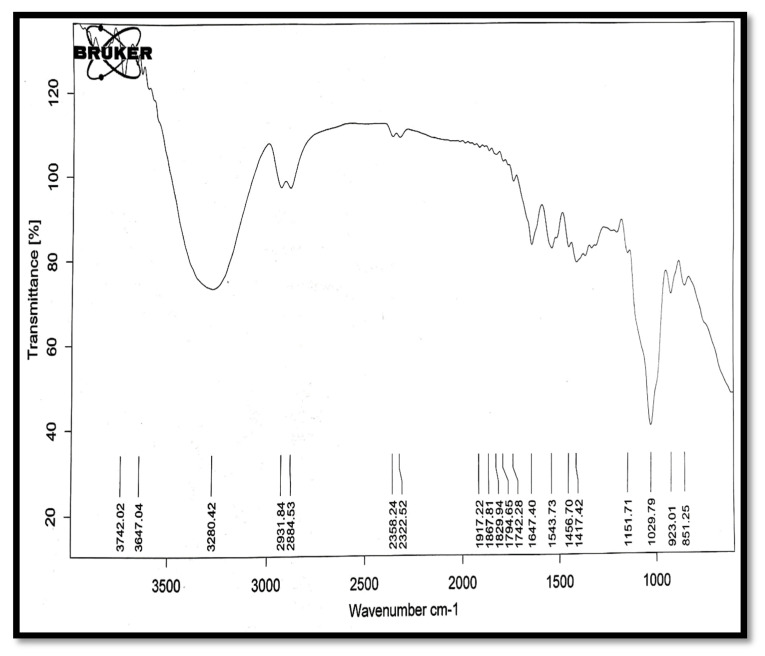
The IR spectrum of the film F1.

In the IR spectrum of the fabricated transdermal film F1 (Figure 12), peaks of C≡N stretch (1544 cm^−1^), O–H stretch (3647 cm^−1^), C–O (1029 cm^−1^), and C=C stretch (1647 cm^−1^) were present. No extra peaks were observed in the IR spectrum of the film, which showed that the components of the formulation were not interacting with each other.

### 3.4. Folding Endurance

All the films prepared demonstrated >300 folding durability, as shown in Table 2.

### 3.5. Film Thickness

The film thickness was determined to vary from 1.00 ± 0.001 to 1.04 ± 0.03 mm. F5 had the highest thickness, and F3 had the lowest. In F5, the quantity of TGP was 200 mg and the quantity of Ct was 100 mg, while in F3, the quantity of Ct was 100 mg and the quantity of TGP was 50 mg [39].

### 3.6. Surface pH

Thakur et al. have prepared transdermal composite films of chitosan–montmorillonite for the delivery of curcumin. Their pH was lower that the skin pH range, i.e., between 7.3 and 7.4 [40]. The results of our experiment were in accordance with this result. The films had a surface pH from 5.7 ± 0.001 (F8) to 6.7 ± 0.001 (F1). The formulation F1 had the highest pH on the surface, while F8 had the lowest.

### 3.7. Weight Variance

Thakur et al. have prepared transdermal composite films of chitosan–montmorillonite for the delivery of curcumin. They found that the film weight variance ranged between 0.36 and 0.37 gm which in accordance with our present results [40]. The film weight variance was between 0.34 ± 0.002 (F6) and 0.47 ± 0.003 gm (F8). F8 had the largest difference in weight and F6 has the lowest. 

### 3.8. Drug Content

Thakur et al. have found drug content ranging between 98.81% and 99.69%, which is also in accordance with our results [40]. Drug content is defined as the total amount of the drug present in the formulation. The drug level of the prepared films was between 96.0 ± 1.43 (F1) and 98.0 ± 1.33 (F6) μg [40]. The highest drug content was found in the F6 batch and the lowest in F1.

### 3.9. Moisture Intake

Thakur et al. have found moisture content between 3.08% and 2.44%, which is in accordance with our results [40]. The intake of moisture (%) in films ranged between 2.83 ± 0.002 for F1 batch and 3.76 ± 0.001 for F8 batch. For the long-term durability of the transdermal films, low moisture accumulation is beneficial and prevents the formulations from microbial contamination. F8 showed the highest drug content and F1 the lowest.

### 3.10. Moisture Uptake

Analysis of moisture uptake showed that a rise in the hydrophilic polymer concentration was directly proportional to an increase in the moisture uptake by the films. Thakur et al. have found the moisture absorption between 8.65% and 6.16%, which is in accordance with our results [40]. The moisture absorption (%) of the films ranged from 5.33 ± 0.22 (F1) to 10.02 ± 0.61 (F8). 

Thakur et al. showed that chitosan–montmorillonite K films swelled very little at an almost neutral pH, compared to a pronounced swelling at a low pH. Therefore, it can be assumed that the Ct-TGP PEC film we fabricated will show very little swelling when applied over skin for transdermal protein delivery. When a fabricated film is applied over skin, a lower skin moisture also limits skin swelling [40].

### 3.11. Surface Morphology

**Figure 13:**
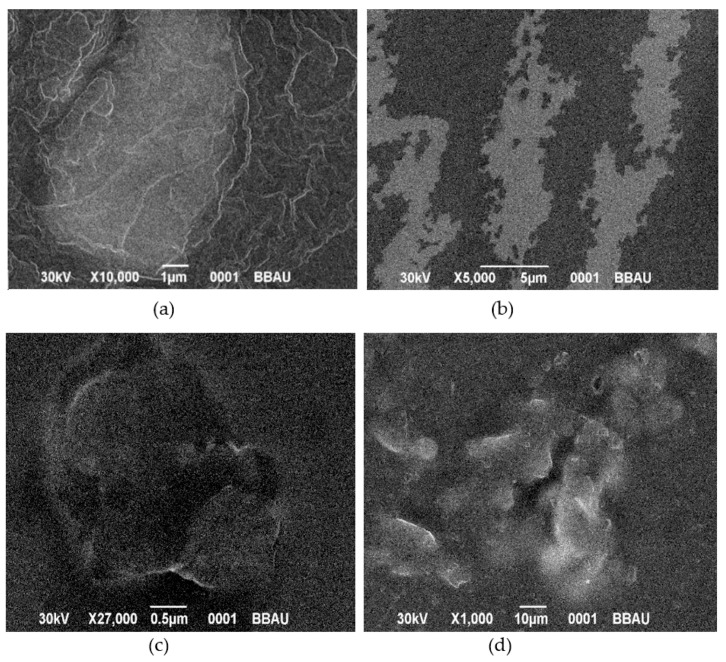
SEM images of films. (**a**) SEM image of a film before drug release study (F1), (**b**) SEM image of a film after drug permeation study (F1), (**c**) SEM image of a film after drug release study (F1) and (**d**) SEM image of a film after five days of drug release study (F1).

SEM (Figure 13a) revealed a smooth film surface, while minor cracks were found after permeation tests (Figure 13b). Film rapturing also occurred during and after drug release analysis (Figure 13c,d, respectively) 

### 3.12. Drug Release 

**Figure 14:**
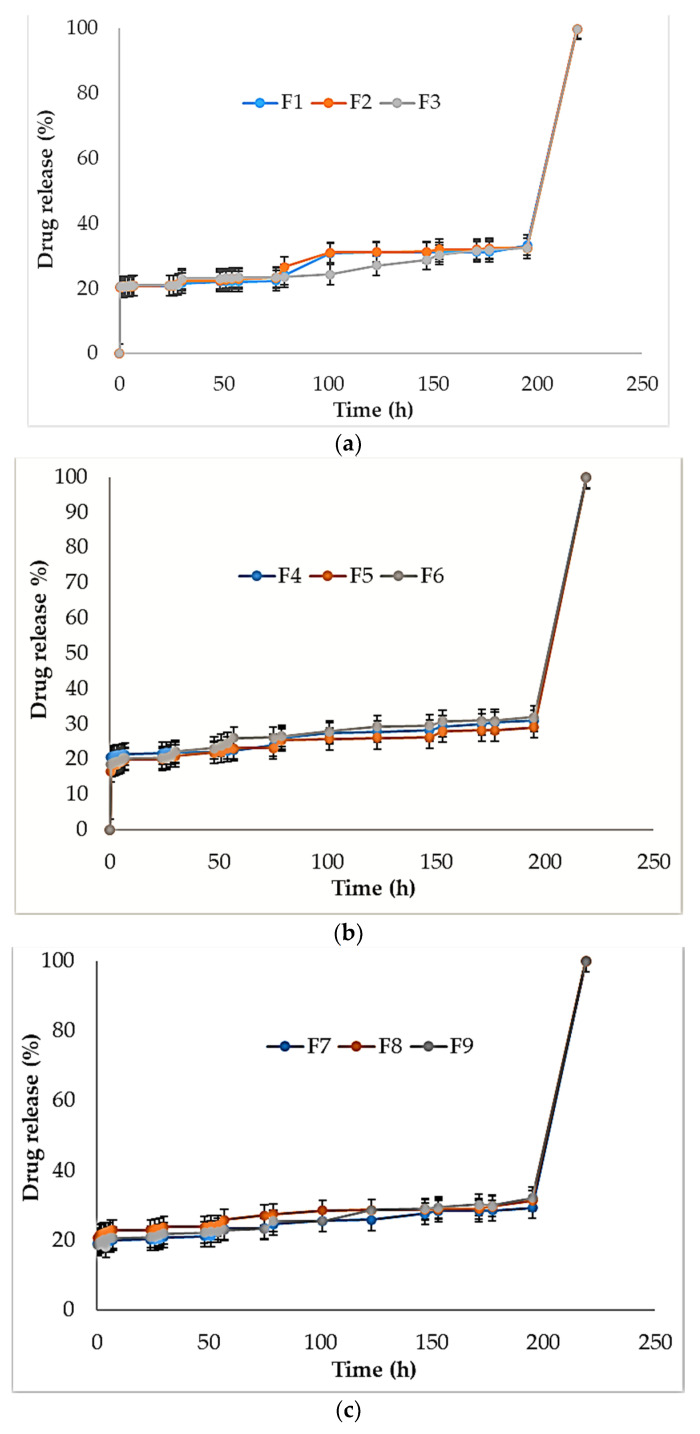
Drug release study of formulations F1–F3 (**a**), F4–F6 (**b**), and F7–F9 (**c**).

The drug release data for all the formulations of films (F1–F9) are shown in Figure 14. Table 3 summarizes the drug release and drug permeation data of fabricated films.

In a study, chitosan–alginate composite films were fabricated and able to release a drug during up to 72 h [7]. In our experiments, during the first 4 days, drug release (%) was between 13.75 ± 1.64 (F2) and 22.54 ± 1.34 (F5) and from day 5 to day 6, it was between 72.67 ± 2.13 (F3) and 78.33 ± 3.13 (F1). Drug permeation (%) during the first 4 days was between 15.78 ± 1.23 and 22.49 ± 1.29.

The T50% (h) and T80%(h) we found in drug release and permeation studies are shown in Table 4. The drug release for T50% was 200–180 h (F1–F9) and for T80%, it was 210–190 h (F1–F9). Drug permeability for T50% was 200–201 h (F1–F9) and for T80%, it was 210–211 h (F1–F9).

### 3.13. Kinetics of Drug Release

#### 3.13.1. Drug Release Kinetics

We found that the formulations adopted the Hixson–Crowell paradigm in their kinetics. The Hixson–Crowell model assumed that the drug’s matrix surface area and diameter changed over time. Results obtained from in vitro drug release experiments using the Hixson–Crowell model were plotted as drug cube root versus time. Equation (11) can be used to evaluate the Hixson–Crowell rate constant.

W_0_ ^1⁄3^ − W_t_^1⁄3^ = K_HC_t (11)

where

W_0_ is the initial amount of drug in the dosage form
W_t_ is the remaining amount of drug in the dosage form at time t
K_HC_t is a constant incorporating the surface–volume relation.

For all medication dosages, a Hixson–Crowell model exists, where the dissolution happens in planes parallel to the drug surface. Formulation measurements decrease proportionally, in such a way that the original geometric shape stays static all the time. The kinetics of drug release of the formulations F1–F9 is shown in Table 5. Based on the data, we demonstrated that the drug release of all the formulations followed the Hixson–Crowell kinetics.

A study on formulation and evaluation of transdermal composites and films of chitosan and montmorillonite for the delivery of curcumin via transdermal route found that there were approximately the same release patterns according to Hixon–Crowell kinetics [40]. Another study was performed on chitosan-based drug delivery via transdermal route to overcome the skin barrier and it was concluded that the regression coefficient (r^2^) was nearly similar to the one found in this study [41].

#### 3.13.2. In Vitro Skin Permeation Studies

**Figure 15:**
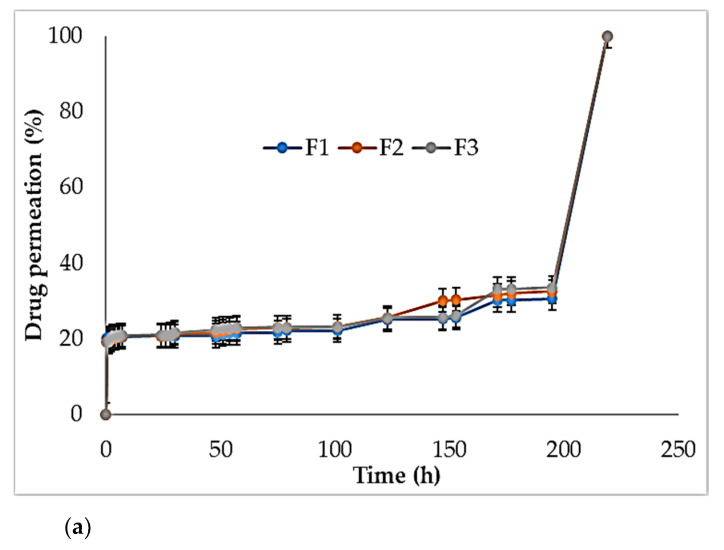

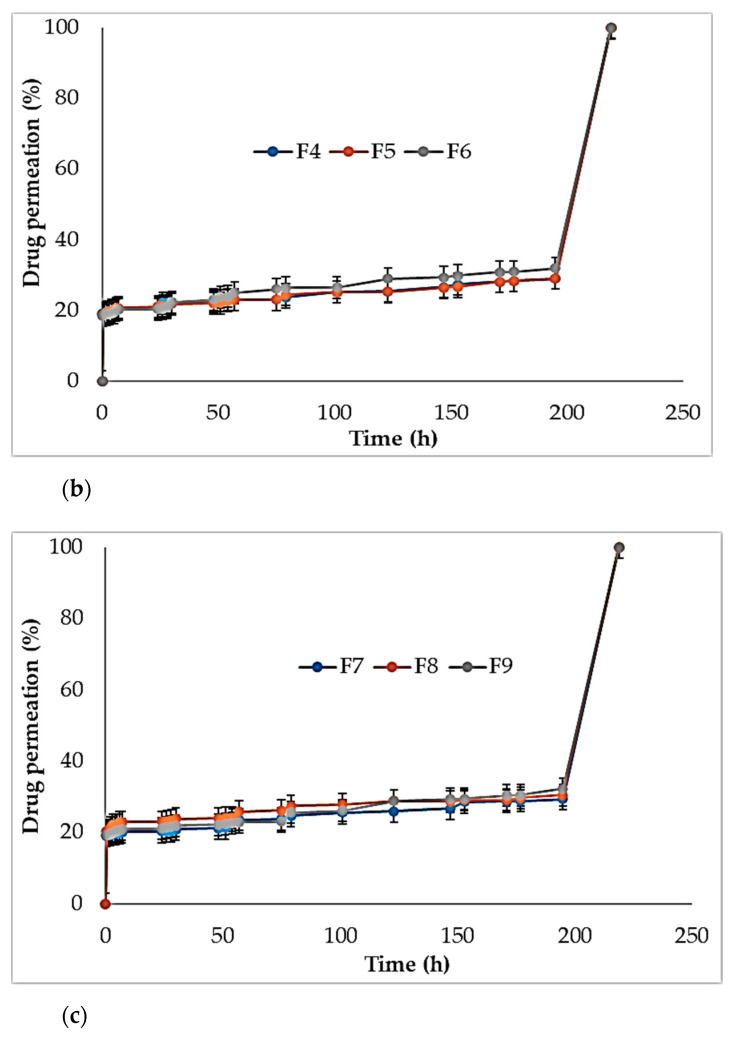
Drug permeation study of F1–F3 (**a**), F4–F6 (**b**), and F7–F9 (**c**).

Results of the drug permeation study are shown in Figure 15.

Thakur et al. prepared chitosan–montmorillonite K transdermal films and found that the fabricated films showed better cellular adhesion and proliferation than a native polymer [40].

#### 3.13.3. Kinetics of Drug Permeation 

Kinetics of drug permeation of F1–F9 are shown in Table 6. Based on the data, we demonstrated that the drug permeation followed the Hixson–Crowell kinetics.

Other authors found that kinetics of permeation of a chitosan-based drug delivery system for the delivery of curcumin was approximately similar to the one investigated in this study. Another study, aimed to overcoming skin barriers in the delivery of drugs through transdermal route, found similar patterns of drug permeation in the skin. 

## 4. Conclusions

Transdermal films of chitosan-based polysaccharide composite films were successfully prepared. The whole film structure was opaque and translucent. The films demonstrated folding durability >300. The film thickness varied between 1.00 ± 0.001 and 1.04 ± 0.03 mm. The formulation F1 had the highest pH on the surface, while F8 had the lowest. The drug release for T50% was 200–180 h and for T80%, it was 210–190 h. Drug permeability for T50% was 200–201 h and for T80%, it was 210–211 h. The formulations adopted the Hixson–Crowell paradigm in kinetics of drug release and permeation.

Such structures can be classified according to their wide range of properties thanks to which they can provide multifunctional materials. Even though natural polysaccharides might be often dissoluble in normal solvents that will limit self-ability to process; nevertheless, other media, such as ionic solvents, can ease the dissolution of polysaccharides.

After establishing the optimal concentration of chitosan and TSP, the selection of a suitable penetration enhancer to improve the bioavailability of peptide molecules in the formulation becomes a critical parameter and it will definitely intensify the therapeutic potential of a drug. Dose adjustment and formulation characteristics should be taken into consideration for a successful development of a transdermal film.

## Figures and Tables

**Table 1:** Quantities of TGP and Ct used for the preparation of films.

S. No.	TGP (mg)	Ct (mg)
1.	100	150
2.	100	100
3.	100	50
4.	200	150
5.	200	100
6.	200	50
7.	150	150
8.	150	100
9.	150	50

**Table 2:** Folding endurance of formulations.

Formulation	Folding Endurance	Thickness (mm)	pH	Weight Variation (gm)	Drug Content (%)	Moisture Intake (%)	Moisture Uptake (%)
F1	>300	1.03 ± 0.001	6.7 ± 0.001	0.35 ± 0.003	96.0 ± 1.43	2.83 ± 0.002	5.33 ± 0.24
F2	>300	1.02 ± 0.001	6.2 ± 0.001	0.36 ± 0.001	96.8 ± 1.64	3.12 ± 0.001	8.12 ± 0.31
F3	>300	1.00 ± 0.011	6.1 ± 0.001	0.39 ± 0.002	97.4 ± 0.89	3.21 ± 0.002	7.21 ± 0.60
F4	>300	1.02 ± 0.001	5.8 ± 0.001	0.41 ± 0.002	97.7 ± 1.02	3.45 ± 0.003	5.45 ± 0.46
F5	>300	1.04 ± 0.001	6.0 ± 0.001	0.38 ± 0.003	96.5 ± 1.86	3.38 ± 0.002	6.38 ± 0.22
F6	>300	1.03 ± 0.001	6.2 ± 0.001	0.34 ± 0.002	98.0 ± 1.33	2.94 ± 0.001	7.94 ± 0.63
F7	>300	1.02 ± 0.067	6.5 ± 0.001	0.40 ± 0.003	97.3 ± 0.98	3.30 ± 0.001	7.30 ± 0.37
F8	>300	1.01 ± 0.031	5.7 ± 0.001	0.47 ± 0.001	96.3 ± 2.03	3.76 ± 0.001	10.02 ± 0.58
F9	>300	1.08 ± 0.033	6.3 ± 0.001	0.42 ± 0.002	97.4 ± 0.87	3.42 ± 0.002	7.44 ± 0.32

**Table 3:** Drug release and drug permeation of formulations.

Formulation	% Drug Release (before 5 Days)	% Drug Release (between 5 to 6 Days)	% Drug Permeation (before 5 Days)	% Drug Permeation (between 5 to 6 Days)
F1	15.87 ± 1.34	78.33 ± 3.13	15.78 ± 1.23	77.93 ± 3.20
F2	13.75 ± 1.64	73.78 ± 4.23	12.73 ± 1.54	72.69 ± 4.21
F3	21.65 ± 2.54	72.67 ± 2.13	20.55 ± 2.49	71.93 ± 2.08
F4	17.89 ± 2.42	73.52 ± 3.53	16.92 ± 2.38	71.49 ± 3.21
F5	22.54 ± 1.34	74.45 ±3.63	22.49 ± 1.29	74.39 ± 2.11
F6	19.28 ± 1.44	74.56 ± 2.84	19.21 ± 1.12	74.47 ± 1.96
F7	18.33 ± 1.75	78.32 ± 4.75	17.15 ± 1.44	77.331 ± 4.62
F8	20.5 ± 1.38	76.29 ± 3.12	18.22 ± 1.16	73.46 ± 3.53
F9	16.4 ± 1.67	77.53 ± 2.11	21.42 ± 1.55	75.18 ± 2.16

**Table 4:** The T50% and T80% of drug release and permeation studies.

Formulation	Time (Drug Release)		Time (Permeation)	
	T_50%_ (h)	T_80%_(h)	T_50%_(h)	T_80%_ (h)
F1	200	210	200	210
F2	200	210	200	210
F3	200	210	200	210
F4	181	190	201	211
F5	200	210	201	211
F6	200	210	200	210
F7	201	211	201	211
F8	201	211	201	211
F9	180	190	200	210

**Table 5:** Kinetics of drug release of formulations F1–F9.

Formulation	Kinetics												
	Zero Order Kinetics		First Order Kinetics		Higuchi Kinetics		Baker Lonsdale Kinetics		Hixson–Crowell Kinetics		Kosermeyer–Peppas Kinetics		
	*R* ^2^	K_0_	*R* ^2^	K_0_	*R* ^2^	K_0_	*R* ^2^	K_0_	*R* ^2^	K_0_	*R* ^2^	K_0_	n
F1	0.478	0.161	0.304	0.006	0.393	1.300	0.907	0.255	0.899	0.001	0.612	5.670	0.090
F2	0.485	0.161	0.304	0.006	0.405	1.306	0.907	0.255	**0.930**	0.001	0.669	5.656	0.095
F3	0.443	0.153	0.279	0.006	0.360	1.317	0.903	0.256	**0.958**	0.001	0.656	5.487	0.079
F4	0.419	0.150	0.264	0.006	0.342	1.328	0.924	0.213	**0.964**	0.001	0.668	5.365	0.073
F5	0.419	0.150	0.698	0.022	0.352	1.277	0.924	0.213	**0.948**	0.001	0.855	6.008	0.089
F6	0.547	0.176	0.698	0.022	0.467	1.265	0.907	0.232	0.880	0.001	0.747	6.214	0.123
F7	0.414	0.148	0.698	0.022	0.340	1.297	0.909	0.239	**0.968**	0.001	0.739	5.735	0.079
F8	0.395	0.142	0.698	0.022	0.333	1.375	0.914	0.261	**0.935**	0.000	0.799	4.984	0.067
F9	0.452	0.155	0.698	0.022	0.934	1.392	0.912	0.235	**0.975**	0.001	0.777	5.793	0.090

**Table 6:** Kinetics of drug permeation (F1–F9).

Formulation	Kinetics												
	Zero Order Kinetics		First Order Kinetics		Higuchi Kinetics		Baker Lonsdale Kinetics		Hixson–Crowell Kinetics		Kosermeyer–Peppas Kinetics		
	*R* ^2^	K_0_	*R* ^2^	K_0_	*R* ^2^	K_0_	*R* ^2^	K_0_	*R* ^2^	K_0_	*R* ^2^	K_0_	n
F1	0.393	0.144	0.258	0.004	0.308	1.309	0.904	0.251	**0.872**	0.000	0.511	5.938	0.061
F2	0.456	0.157	0.297	0.004	0.369	1.291	0.909	0.240	**0.939**	0.001	0.634	5.717	0.084
F3	0.437	0.153	0.281	0.004	0.352	1.300	0.911	0.240	**0.859**	0.001	0.604	5.584	0.077
F4	0.391	0.143	0.256	0.004	0.323	1.312	0.908	0.239	**0.978**	0.000	0.844	5.579	0.073
F5	0.390	0.143	0.257	0.004	0.321	1.309	0.909	0.237	**0.979**	0.000	0.825	5.584	0.073
F6	0.471	0.159	0.308	0.004	0.403	1.285	0.908	0.232	**0.970**	0.001	0.844	5.997	0.104
F7	0.414	0.148	0.277	0.004	0.340	1.294	0.907	0.238	**0.974**	0.001	0.755	5.767	0.080
F8	0.392	0.141	0.234	0.004	0.331	1.367	0.913	0.255	**0.938**	0.000	0.84	5.046	0.068
F9	0.451	0.155	0.292	0.004	0.373	1.297	0.907	0.240	**0.980**	0.001	0.750	5.727	0.088

## Data Availability

The data presented in this study are available on reasonable request from the corresponding author.

## References

[1] Keservani R.K., Sharma A.K., Jarouliva U. (2015). Protein in Drug Targeting and its Therapeutic Approach. Ars. Pharm..

[2] Bruno B.J., Miller G.D., Lim C.S. (2013). Basics and recent advances in peptide and protein drug delivery. Ther. Deliv..

[3] Alkilani A.Z., McCrudden M.T.C., Donnelly R.F. (2015). Transdermal Drug Delivery: Innovative Pharmaceutical Developments Based on Disruption of the Barrier Properties of the stratum corneum. Pharmaceutics.

[4] Pawłowska S., Rinoldi C., Nakielski P., Ziai Y., Urbanek O., Li X., Kowalewski T.A., Ding B., Pierini F. (2020). Ultraviolet Light-Assisted Electrospinning of Core–Shell Fully Cross-Linked P(NIPAAm-co-NIPMAAm) Hydrogel-Based Nanofibers for Thermally Induced Drug Delivery Self-Regulation. Adv. Mater. Interfaces.

[5] Nakielski P., Pawłowska S., Rinoldi C. (2020). Multifunctional Platform Based on Electrospun Nanofibers and Plasmonic Hydrogel: A Smart Nanostructured Pillow for Near-Infrared Light-Driven Biomedical Applications. ACS Appl. Mater. Interfaces.

[6] El-Boubbou K., Huang X. (2011). Glyco-nanomaterials: Translating insights from the “sugar-code” to biomedical applications. Curr. Med. Chem..

[7] Zheng Y., Monty J., Linhardt R.J. (2015). Polysaccharide-based nanocomposites and their applications. Carbohydr. Res..

[8] Suh J.-K.F., Matthew H.W. (2000). Application of chitosan-based polysaccharide biomaterials in cartilage tissue engineering: A review. Biomaterials.

[9] Liu L., Liu L., Liu C.-K., Fishman M.L., Hicks K.B. (2007). Composite films from pectin and fish skin gelatin or soybean flour protein. J. Agric. Food Chem..

[10] Dumitriu S. (2004). Polysaccharides: Structural Diversity and Functional Versatility.

[11] Crini G. (2005). Recent developments in polysaccharide-based materials used as adsorbents in wastewater treatment. Prog. Polym. Sci..

[12] Stanton J., Xue Y., Pandher P., Malek L., Brown T., Hu X., Salas-de la Cruz D. (2018). Impact of ionic liquid type on the structure, morphology and properties of silk-cellulose biocomposite materials. Int. J. Biol. Macromol..

[13] Malviya R., Sharma P.K., Dubey S.K. (2020). Efficiency of self-assembled etoricoxib containing polyelectrolyte complex stabilized cubic nanoparticles against human cancer cells. Precis. Med. Sci..

[14] Bansal V., Sharma P.K., Sharma N., Pal O.P., Malviya R. (2011). Application of chitosan and chitosan derivatives in drug delivery. Adv. Biol. Res..

[15] Paluszkiewicz C., Stodolak E., Hasik M., Blazewicz M. (2011). FT-IR study of montmorillonite-chitosan nanocomposite materials. Spectrochim Acta A Mol. Biomol. Spectrosc..

[16] Sharma C., Dinda A.K., Potdar P.D., Mishra N.C. (2015). Fabrication of quaternary composite scaffold from silk fibroin, chitosan, gelatin, and alginate for skin regeneration. J. Appl. Polym. Sci..

[17] Malviya R., Raj S., Fuloria S., Subramaniyan V., Sathasivam K., Kumar U., Unnikrishnan D., Porwal O., Kumar D.H., Singh A. (2021). Evaluation of antitumor efficacy of chitosan-tamarind gum polysaccharide polyelectrolyte complex stabilized nanoparticles of simvastatin. Int. J. Nanomedicine.

[18] Bangale G.S., Stephen R.B., Rajesh K.S., Shinde G.V., Deepak U.G., Rajveer C., Kumaraswamy D., Panicker P.S. (2010). Design and evaluation of transdermal films of Atenolol. J. Chem. Pharm. Res..

[19] Verma P.R.P., Iyer S.S. (2000). Transdermal delivery of propranolol using mixed grades of eudragit, Design and in vitro and in vivo evaluation. Drug Deliv. Indian Pharm..

[20] Lewis S., Pandey S., Udupa N. (2006). Design and evaluation of matrix type and membrane controlled transdermal delivery systems of nicotine suitable for use in smoking cessation. Indian J. Pharm. Sci..

[21] Bharkatiya M., Nema R.K., Bhatnagar M. (2010). Designing and characterization of drug free patches for transdermal application. Int. J. Pharm. Sci. Drug Res..

[22] Costa P., Ferreria D.C., Morgado R., Sousa L.J.M. (1997). Design and evaluation of a lorazepam transdermal delivery system. Drug Deliv. Indian Pharm..

[23] Bagyalakshmi J., Vamsikrishna R.P., Manavalan R., Ravi T.K., Manna P.K. (2007). Formulation development and in vitro and in vivo evaluation of membrane-moderated transdermal systems of ampicillin sodium in ethanol: PH 4.7 buffer solvent system. AAPS Pharm. Sci. Tech..

[24] Aggrawal G., Dhawan S. (2009). Development, fabrication and evaluation of transdermal drug delivery systema review. Pharmainfo.

[25] Keleb E., Sharma R.K., Mosa E.B. (2010). Transdermal drug delivery system-design and evaluation. Int. J. Adv. Pharm. Sci..

[26] Raghuram R.K., Muttalik S., Reddy S. (2003). Once-daily sustained release matrix tablets of nicorandil: Formulation and in-vitro evaluation. AAPS Pharm. Sci. Tech..

[27] Shah V.P., Lesko L.J., Fan J., Fleischer N., Handerson J., Malinowski H., Makary M., Ouderkirk L., Roy S., Sathe P. (1997). FDA guidance for industry: Dissolution testing of immediate release solid oral dosage forms. Dissolution Technol..

[28] Crank J. (1975). The Mathematics of Diffusion.

[29] Arhewoh M.I., Okhamafe O.A. (2004). An overview of site-specific delivery of orally administered proteins/peptides and modelling considerations. J. Med. Biomed. Res..

[30] Hixson A.W., Crowell J.H. (1931). Dependence of Reaction Velocity upon surface and Agitation. Ind. Eng. Chem..

[31] Rao P.R., Diwan P.V. (1998). Formulation and in vitro evaluation of polymeric films of diltiazem hydrochloride and indomethacin for transdermal administration. Drug Dev. Ind. Pharm..

[32] Kumar De P., Mallick S., Mukherjee B., Sengupta S., Pattnaik S., Chakraborty S. (2011). Optimization of In-Vitro Permeation Pattern of Ketorolac Tromethamine Transdermal Patches. Iran J. Pharm. Res..

[33] Sadeq Z.A., Rajab N.A., Abd Alhammid S.N., Zaki H. (2019). Preparation, in-vitro Evaluation, Mechanical Characterization, and Release Of Nebivelol Hydrochloride as A Transdermal Film using combined Eudragite-Polyvinyl Alcohol as Adhesive Film Forming Polymer. J. Pharm. Sci. Res..

[34] Kaur R., Sharma A., Puri V., Singh I. (2019). Preparation and characterization of biocomposite films of carrageenan/locust bean gum/montmorrillonite for transdermal delivery of curcumin. BioImpacts.

[35] Martins J.T., Souza W.S., Vicente A.A. (2013). Biocomposite films based on κ-carrageenan/locust bean gum blends and clays: Physical and antimicrobial properties. Food Bioprocess Tech..

[36] Salcedo I., Aguzzi C., Sandri G., Bonferoni M.C., Mori M., Cerezo P., Sánchez R., Viseras C., Caramella C. (2012). In vitro biocompatibility and mucoadhesion of montmorillonite chitosan nanocomposite: A new drug delivery. Appl. Clay Sci..

[37] Depan D., Kumar A.P., Singh R.P. (2009). Cell proliferation and controlled drug release studies of nanohybrids based on chitosan-g-lactic acid and montmorillonite. Acta Biomater..

[38] Rao M.R., Sonavane V., Kulkarni S., Magar M., Zope A., Karanjkar P. (2019). Design of transdermal patch of ketoprofen by full factorial design for treatment of rheumatoid arthritis. J. Drug Deliv..

[39] Zhang B.X., Azuma J.I., Uyama H. (2015). Preparation and characterization of a transparent amorphous cellulose film. RSC Adv..

[40] Thakur G., Singh A., Singh I. (2016). Formulation and evaluation of transdermal composite films of chitosan-montmorillonite for the delivery of curcumin. Int. J. Pharm. Investig..

[41] Subramanian K., Indumathi S., Vijayakumar V. (2014). Fabrication and Evaluation of Chitosan-Gelatin Composite Film As a Drug Carrier for in Vitro Transdermal Delivery. Int. J. Pharm. Sci. Res..

